# Protective effects of *Launaea procumbens* on rat testis damage by CCl_4_

**DOI:** 10.1186/1476-511X-11-103

**Published:** 2012-08-22

**Authors:** Rahmat Ali Khan

**Affiliations:** 1 Department of Biotechnology, Faculty of Biological Sciences, University of Science and Technology Bannu, Bannu, KPK, Pakistan

**Keywords:** *Launaea procumbens*, GSH, CCl_4_, Lipidperoxidation, DNA damages

## Abstract

**Background:**

Traditionally various human diseases of kidneys, hormonal imbalance and sexual diseases are treated with *Launaea procumbens* (L). In the present study protective effects of methanolic extract of *Launaea procumbens* (LPME) was evaluated against CCl_4_-induced oxidative damages in rat testis.

**Methods:**

To examine the protective effects of *Launaea procumbens* on testis against oxidative stress of carbon tetrachloride in male rat, 30 male albino rats were equally divided into 5 groups (6 rats). First group was given standard diet and drinking water. Second group received CCl_4_ 3 ml/kg intraperitoneally (30% in olive oil). Third and forth were given orally 100; 200 mg/kg b.w., in 99.8% dimethyl sulphooxide (DMSO), *Launaea procumbens* methanolic extracts (LPME) after 48 h of CCl_4_ treatment twice a week and sixth group received only LPME in DMSO at a dose of 200 mg/kg b.w., for four weeks. Protective effects of *Launaea procumbens* were observed on sperm concentration, motility and morphology, serum reproductive hormonal level, activity of antioxidant enzymes, lipid peroxidation (TBARS) and DNA damages.

**Results:**

Results of the present study revealed that treatment of CCl_4_ significantly *(p < 0.01)* reduced sperm concentration and motility comparatively to controls. Level of testosterone, luteinizing hormone and follicle stimulating hormone, were depleted markedly (*p <0.01*) with treatment of CCl_4_. In addition, CCl_4_ induction in rats reduced activities of antioxidant enzymes while increased lipid peroxidation and DNA damages. Co-administration of LPME significantly (*p <0.01*) improved these alterations in improving of hormonal level, activities of antioxidant enzymes and lipid peroxidation near to control rats.

**Conclusion:**

From the results it is suggested that *Launaea procumbens* methanolic extract has the ability to protect testis against oxidative damages, possibly through antioxidant effects of its bioactive compounds.

## Background

Male sexual dysfunction composed of several problems associated with sperm concentration, motility and hormonal imbalance e.g., low testosterone level, which are caused by alcoholism, drug abuse, aging and cigarette smoking, anti depressant drugs and exposure of toxic chemicals [1-3]. Carbon tetrachloride is an industrial solvent cause kidney, lungs and testicular damages in experimental animals [4] triggers oxidative damages through free radicals produced from CCl_4_ metabolism [5]. Free radicals of CCl_4_ bind with polyunsaturated fatty acid (PUFA) of sperm membrane to produce alkoxy and peroxy radicals that, in turn, generate lipid peroxides, that are highly reactive, change sperm concentration, alters hormonal levels, reduces enzyme activity and finally induce injury or necrosis [6,7]. Free radicals causes reduction in GSH contents and alteration of reproductive hormones, oxidative DNA damages, genetic mutation, DNA strand breakage and chromosomal alterations [8,9], necrosis of spermatocytes/spermatids and degeneration in seminiferous tubules [10,11]. Medicinal plants are also in high demand for application of functional food or biopharmaceuticals because of consumer preferences. Currently various medicinal plants has been investigated based on the integrative approaches on drug development from Ayurveda. Several medicinal plants like *Digera muricata* are used as a potent antioxidant against chemical induced oxidative stress [7].

*Launaea procumbens* is traditionally used in the treatment of rheumatism [12], kidney dysfunction [13], reproductive disorder and hormonal imbalance in male [14,15]. Nutritional analysis showed that *Launaea procumbens* composed of salicylic acid, vanllic acid, synergic acid, 2-methyl-resercinol and gallic acid [16] which has antioxidant, anticancer and anti-inflammatory properties. Therefore the present study was arranged to evaluate the traditional use of methanol extract of *Launaea procumbens* versus carbon tetrachloride induced oxidative stress associated reproductive hormonal changes and lipids peroxidation in rats.

## Materials and methods

### Plant collection and extraction

*Launaea procumbens* at maturity were collected from Wah Cantt District Rawalpindi (Pakistan). Plants were identified and a specimen was submitted at Herbarium of Pakistan, Quaid-i-Azam University Islamabad, Pakistan. Whole plant (leaves, stem, flowers and seeds) were shade dried at room temperature for two weeks, chopped, ground mechanically of mesh size 1 mm. 2 kg powder of *Launaea procumbens* was extracted twice in 5 liter of methanol with random shaking, after a week the extract was filtered through Whatmann filter paper No. 45, filtrate was mixed and evaporated through rotary vacuum evaporator at 40°C to get crude methanolic crude extract (LPME). The crude extract was stored at 4°C for further in vivo investigations [11].

### Animals

Six week old, 30 male albino rats (180–190 g) were provided by National Institute of Health Islamabad and were kept in ordinary cages at room temperature of 25 ± 3°C with a 12 h dark/light cycle. They were allowed to standard laboratory feed and water.

### Ethical approval

The study protocol was approved by Ethical Committee of Quaid-i-Azam University Islamabad.

### Experimental design

To study the antioxidant attributes of *Launaea procumbens*, male albino rats were equally divided into 5 groups (6 rats). Group 1 (control) have free access to food materials. Group II received CCl_4_ 3 ml/kg intraperitoneally (30% in olive oil). Group III and IV were given orally 100; 200 mg/kg b.w. (in DMSO), *Launaea procumbens* methanolic extracts (LPME) after 48 h of CCl_4_ induction in rats, while group V received only LPME in DMSO at a dose of 200 mg/kg b.w., orally. After 24 h of the last treatment, all the animals were weighted and collected their blood for serum hormonal level. Then the testes and epididymis from the rats were carefully dissected and weighed independently. From the epididymis, sperm were collected, mounted on a slide and their motility assessed immediately under the microscope at × 10 objective. The motility assessment was expressed as percentage motile forms. The slides were later stained with Carbol Fuschin and the sperm number and morphology were examined. After the process one testis of each rat was treated with liquid nitrogen for further enzymatic and DNA damage analysis while the other was processed for histology.

### Serum analysis of hormone

Serum level of testosterone, luteinizing hormone (LH) and follicle stimulating hormone (FSH) was estimated using RIA gamma counter through kits (10227-Czch Republic purchased from IMMUNOTECH Company).

### Assessment of antioxidant enzymes

70 mg of tissue was homogenized in 10 volume of 100 mmol KH_2_PO_4_ buffer containing 1 mmol EDTA (pH 7.4) and centrifuged at 12,000 × g for 30 min at 4°C. The supernatant was collected and used for enzymatic studies. Protein concentration of tissue supernatant was determined by the method of Lowry et al. [17] using crystalline BSA as standard.

### Catalase assay (CAT)

CAT activities were determined by the method of Chance and Maehly [18]. The reaction solution of CAT activities contained: 2.5 ml of 50 mmol phosphate buffer (pH 5.0), 0.4 ml of 5.9 mmol H_2_O_2_ and 0.1 ml enzyme extract. Changes in absorbance of the reaction solution at 240 nm were determined after one minute. One unit of CAT activity was defined as an absorbance change of 0.01 as units/min.

### Peroxidase assay (POD)

Chance and Maehly [18] protocol were used determination of POD activities. 3 ml reaction solution of POD contained 0.1 ml enzyme extract, 2.5 ml 50 mM phosphate buffer (pH 5.0), 0.1 ml of 20 mM guaiacol, and 0.3 ml H_2_O_2_ (40 mM). Measure absorbance changes at 470 nm after one minute and POD activity.

### Superoxide dismutase assay (SOD)

SOD activity was estimated by the method of Kakkar et al. [19]. Reaction mixture of this method contained: 0.1 ml of phenazine methosulphate (186 μmol), 1.2 ml of sodium pyrophosphate buffer (0.052 mmol; pH 7.0), 0.3 ml of supernatant after centrifugation (1500 x g for 10 min followed by 10000 x g for 15 min) of homogenate was added to the reaction mixture. Enzyme reaction was initiated by adding 0.2 ml of NADH (780 μmol) and stopped after 1 min by adding 1 ml of glacial acetic acid. Amount of chromogen formed was measured by recording color intensity at 560 nm. Results are expressed in units/mg protein.

### Estimation of lipid peroxidation assay (TBARS)

The assay for lipid peroxidation was carried out by the modified method of Iqbal et al. [20]. The reaction mixture in a total volume of 1.0 ml contained 0.58 ml phosphate buffer (0.1 mol; pH 7.4), 0.2 ml homogenate sample, 0.2 ml ascorbic acid (100 mmol), and 0.02 ml ferric chloride (100 mmol). The reaction mixture was incubated at 37°C in a shaking water bath for 1 h. The reaction was stopped by addition of 1.0 ml 10% trichloroacetic acid. Following addition of 1.0 ml 0.67% thiobarbituric acid, all the tubes were placed in boiling water bath for 20 min and then shifted to crushed ice-bath before centrifuging at 2500 × g for 10 min. The amount of TBARS formed in each of the samples was assessed by measuring optical density of the supernatant at 535 nm using spectrophotometer against a reagent blank. The results were expressed as nmol TBARS/min/mg tissue at 37°C using molar extinction coefficient of 1.56 × 10^5^ M^-1^ cm^-1^.

### Glutathione-S-transferase assay (GST)

Glutathione-S-transferase activity was assayed by the method of Habig et al. [21]. The reaction mixture consisted of 1.475 ml phosphate buffer (0.1 mol, pH 6.5), 0.2 ml reduced glutathione (1 mmol), 0.025 ml (CDNB) (1 mmol) and 0.3 ml of homogenate in a total volume of 2.0 ml. The changes in the absorbance were recorded at 340 nm and enzymes activity was calculated as nmol CDNB conjugate formed/min/mg protein using a molar extinction coefficient of 9.6 × 10^3^ M^-1^ cm^-1^.

### Glutathione reductase assay (GSR)

Glutathione reductase activity was determined by method of Carlberg and Mannervik [22]. The reaction mixture consisted of 1.65 ml phosphate buffer: (0.1 mol; pH 7.6), 0.1 ml EDTA (0.5 mmol), 0.05 ml oxidized glutathione (1 mmol), 0.1 ml NADPH (0.1 mmol) and 0.1 ml of homogenate in a total volume of 2 ml. Enzyme activity was quantitated at 25°C by measuring disappearance of NADPH at 340 nm and was calculated as nmol NADPH oxidized/min/mg protein using molar extinction coefficient of 6.22 × 10^3^ M^-1^ cm^-1^.

### Glutathione peroxidase assay (GSH-Px)

Glutathione peroxidase activity was assayed by the method of Mohandas et al. [23]. The reaction mixture consisted of 1.49 ml phosphate buffer (0.1 mol; pH 7.4), 0.1 ml EDTA (1 mmol), 0.1 ml sodium azide (1 mmol), 0.05 ml glutathione reductase (1 IU/ml), 0.05 ml GSH (1 mmol), 0.1 ml NADPH (0.2 mmol), 0.01 ml H_2_O_2_ (0.25 mmol) and 0.1 ml of homogenate in a total volume of 2 ml. The disappearance of NADPH at 340 nm was recorded at 25°C. Enzyme activity was calculated as nmol NADPH oxidized/min/mg protein using molar extinction coefficient of 6.22 × 10^3^ M^-1^ cm^-1^.

### Reduced glutathione assay (GSH)

Reduced glutathione was estimated by the method of Jollow et al. [24]. 1.0 ml sample of homogenate was precipitated with 1.0 ml of (4%) sulfosalicylic acid. The samples were kept at 4°C for 1 h and then centrifuged at 1200 × g for 20 min at 4°C. The total volume of 3.0 ml assay mixture contained 0.1 ml filtered aliquot, 2.7 ml phosphate buffer (0.1 mol; pH 7.4) and 0.2 ml DTNB (100 mmol). The yellow color developed was read immediately at 412 nm on a SmartSpecTM plus Spectrophotometer. It was expressed as μmol GSH/g tissue.

### DNA fragmentation% assay

DNA fragmentation % assay was conducted using the procedure of Wu et al. [25] with some modifications. The tissue (50 mg) was homogenized in 10 volumes of a TE solution pH 8.0 (5 mmol Tris–HCl, 20 mmol EDTA) and 0.2% triton X-100. 1.0 ml aliquot of each sample was centrifuged at 27,000 × g for 20 min to separate the intact chromatin (pellet, B) from the fragmented DNA (supernatant, T). The pellet and supernatant fractions were assayed for DNA content using a freshly prepared DPA (Diphenylamine) solution for reaction. Optical density was read at 620 nm with (SmartSpecTM Plus Spectrophotometer catalog # 170–2525) spectrophotometer. The results were expressed as amount of % fragmented DNA by the following formula;

(1) %Fragmented DNA = T x 100/T + B

### DNA ladder assay

DNA was isolated using proteinase K and RNase A with the methods of Gilbert et al. [26] to estimate DNA damages. 5 μg of rat DNA was separately loaded in 1.5% agarose gel containing 1.0 μg/ml ethidium bromide including DNA standards (0.5 μg per well). Electrophoresis was performed for 45 min at 100 Volt. After electrophoresis gel was studied under gel doc system and was photographed through digital camera.

### Histopathological overview of testis

After weighting the tissue for histology, testis were placed for 3–4 hrs in formalin and transferred in cedar wood oil. After 72 h treatment testis were shifted in paraplast and prepared blocks for further microtomy. 3–4 μm thin slides were prepared with microtome; wax was removed, stained with hemotoxilin-eosin and photographed under light microscope at 40x.

### Statistical analysis

Data were expressed as mean and standard error (SE) and ANOVA test was used to analyze the difference among various treatments, with least significance difference (LSD) at 0.05 and 0.01 as a level of significance. SPSS ver. 14.0 (Chicago, IL, USA) and Microsoft Excel 2007 (Roselle, IL, USA) were used for the statistical and graphical evaluations.

## Results

### Effect of LPME on sperm parameters

Effects of CCl_4_ on the sperm count and motility was significantly reduced *(p < 0.01)* while the percentage sperm abnormality was significantly *(p < 0.01)* increased after treatment with CCl_4_ comparatively to controls (Table 1). Administration of LPME in CCl_4_ treated rats significantly *(p < 0.01)* attenuated the spermatic alterations as compare to control. There was a significant decrease *(p < 0.01)* in sperm abnormal morphology in CCl_4_ treated rats, the percentage abnormal morphology in comparison with CCl_4_ treated rats. No significant *(p < 0.01)* changes was observed in rats treated with 200 mg/kg LPME alone.

**Table 1:** Effect of LPME on sperm count, motility and morphology male in rat

**Treatment**	**Count (10**^**6**^**/ml)**	**Motility (%)**	**Morphology (%)**
Control	32.1 +/− 2.78++	82.3 +/− 5.**0**++	11.5 +/− 0.95++
3 ml/kg CCl_4_	15.5 +/− 0.54**	52.5+/− 2.9**	35.0 +/− 0.57**
100 mg/kg LPME + CCl_4_	27.0 +/− 0.48*++	70.6+/− 3.3*++	23.1 +/− 0.29*++
200 mg/kg LPME + CCl_4_	31.6 +/− 1.85++	77.5 +/− 1.3++	14.5 +/− 1.39++
200 mg/kg LPME alone	35.7 +/− 2.04++	84.2 +/− 2.0++	11.3+/− 1.14++

*, ** indicate significance from the control group at P < 0.05 and P < 0.01 probability level, respectively.

++ indicate significance from the CCl_4_ group at P < 0.01 probability level, respectively.

### Effect of LPME on pituitary–gonadal axis

The effects of LPME on testosterone, luteinizing hormone and follicle stimulating hormone are shown in Table 2. CCl_4_ administration in rats for 4 weeks, significantly decreased *(p < 0.01)* the hormonal level of testosterone, luteinizing hormone and follicle stimulating hormone comparatively to control group. Alterations of these hormones was significantly reversed (*p < 0.01)* by administrations of 100 mg/kg b.w., and 200 mg/kg and 200 mg/kg b.w., LPME in CCl_4_ treated rats however, non significant *(p > 0.05)* changes were observed in non treated LPME alone rats.

**Table 2:** Effect of LPME on FSH, LH, testosterone, prolactin and estradiol in rat

**Treatment**	**FSH (mg/dl)**	**LH (mg/dl)**	**Testosterone (mg/dl)**	**Prolactin (mg/dl)**	**Estradiol (mg/dl)**
Control	10.3 +/− 0.65++	32 +/− 1.12++	47.8 +/− 2.6++	14 +/− 1.42++	24.5 +/− 2.65++
3 ml/kg CCl_4_	4.9 +/− 0.82**	14.8 +/− 2.51**	25.7 +/− 3.6**	33.6 +/− 2.6**	60.3 +/− 2.8**
100 mg/kg LPME + CCl_4_	9.1 +/− 0.54++	31.9 +/− 3.04++	42.0 +/− 2.09++	16.0 +/− 2.02++	30.5.3 +/− 3.5++
200 mg/kg LPME + CCl_4_	11.5 +/− 0.57++	31.4 +/− 2.87++	44.5 +/− 3.50**++	16.5 +/− 3.21*++	32.4 +/− 2.8++
200 mg/kg LPME alone	11.5 +/− 0.58++	33.3 +/− 3.12++	46.5 +/− 4.20++	13.50 +/− 1.51++	23.6 +/− 1.67++

Mean ± SE (n = 6 number).

*, ** indicate significance from the control group at *P <* 0.05 and *P <* 0.01 probability level.

++ indicate significance from the CCl_4_ group at *P <* 0.01 probability level.

### Effect of LPME on antioxidant profile

Antioxidant profile play important role in infertility. The effects of LPME against CCl_4_ induced antioxidant status alteration are shown in Table 3. Activities of antioxidant enzymes such as CAT, POD and SOD were significantly *(p < 0.01)* reduced by treatment of CCl_4_ as compared to control group. This reduction was improved significantly *(P < 0.01)* by post-administration of LPME at both 100 mg/kg and 200 mg/kg body weight near to control rat. However, non significant changes *(p > 0.05)* were found by administration of LPME alone against the control group.

**Table 3:** Effect of LPME on testis CAT, POD and SOD activity in rat

**Treatment**	**Protein (μg/mg tissue)**	**CAT (U/min)**	**POD (U/min)**	**SOD (U/mg protein)**
Control	2.01 +/− 0.06++	5.56 +/− 0.32++	6.88 +/− 0.29++	2.07 +/− 0.06++
3 ml/kg CCl_4_	0.91 +/− 0.03**	3.06 +/− 0.14**	3.97 +/− 0.09**	0.97 +/− 0.03**
100 mg/kg LPME + CCl_4_	1.86 +/− 0.00++	4.73 +/− 0.14++	4.81 +/− 0.09++	1.35 +/− 0.01++
200 mg/kg LPME + CCl_4_	2.00 +/− 0.07++	5.69 +/− 0.30 ++	6.27 +/− 0.29++	2.00 +/− 0.02++
200 mg/kg LPME alone	2.11 +/− 0.05 ++	5.73 +/− 0.32++	6.98 +/− 0.27++	2.17 +/− 0.05 ++

Mean ± SE (n = 6 number).

*, ** indicate significance from the control group at *P <* 0.05 and *P <* 0.01 probability level.

++ indicate significance from the CCl_4_ group at *P <* 0.01 probability level.

### Effect of LPME on GSHpx, GST, GSR, GSH, TBARS

Effect of CCl_4_ and the protective effects of LPME on tissue phase II metabolizing enzymes viz; GSH-Px, GST, GSR, GSH and TBARS are shown in Table 4. CCl_4_ treatment to rats significantly *(p < 0.01)* decreased the activities of GSH-Px, GST, GSR and GSH while significantly *(p < 0.01)* increased the contents of TBARS in tissue homogenate as compared to control group. 100 mg/kg and 200 mg/kg b.w., LPME showed significant protection and recovered *(p < 0.01)* the activity of enzymes near to control rat; increased the activities of GST, GSR and GSH while decreased the contents of TBARS in a dose dependent manner. LPME when administered alone did not show significant variations.

**Table 4:** Effect of LPME on testis GST, GSH-Px, GSR, GSH and TBARS in rat

**Treatment**	**GSH-Px (nM/mg protein)**	**GSR (nM/min/mg protein)**	**GST (nM/min/mg protein)**	**GSH (μM/g tissue)**	**TBARS (nM/min/mgprotein)**
Control	138.83 +/− 1.0++	201.5 +/−1.93++	84.67 +/− 1.6++	0.89 +/− 0.05 ++	15.3 +/− 0.83++
3 ml/kg CCl_4_	70.50 +/− 0.76**	115.17 +/− 2.2**	55.17 +/− 1.6**	0.60 +/− 0.09**	34.1 +/− 0.44**
100 mg/kg LPME + CCl_4_	129.17 +/− 1.1++	149.83 +/− 2.8++	78.83 +/− 1.3 ++	0.80 +/− 0.06++	21.8 +/− 0.74++
200 mg/kg LPME + CCl_4_	138.33 +/− 1.4++	196.8 +/− 3.2*++	85.17 +/− 2.8 ++	0.81 +/− 0.01**++	17.8 +/− 0.61++
200 mg/kg LPME alone	140.6 +/− ± 1.2++	206.7+/− 2.2++	88.5 +/− 1.9 ++	0.91 +/− 0.01++	15.4 +/− 0.62++

Mean ± SE (n = 6 number).

*, ** indicate significance from the control group at *P <* 0.05 and *P <* 0.01 probability level.

++ indicate significance from the CCl_4_ group at *P <* 0.01 probability level.

### Body weight, testis weight, relative testis weight

Effect of CCl_4_ on body weight, testis weight and relative testis weight are shown in Table 5. CCl_4_ administration to rats significantly increased *(p < 0.01)* testis weight and relative testis weight while significantly decreased *(p < 0.01)* body weight compared to control group. Post-treatment with LPME erased the CCl_4_ toxicity and significantly *(p < 0.01)* improved testis weight and relative testis weight and relative tissue weight towards the control group in a dose dependent However, non significant *(p > 0.05)* variations were observed by LPME alone as compared to control group.

**Table 5:** Effect of LPME on testis weight, relative testis weight in rat

**Treatment**	**Tissue weight (g)**	**Relative testis weight**	**%DNA Fragmentation**
Control	6.07 +/− 0.32++	0.067 +/− 0.003++	5.67 +/− 0.55 ++
3 ml/kg CCl_4_	7.91 +/− 0.21**	0.07 +/− 0.002**	32.67 +/− 1.78**
100 mg/kg LPME + CCl_4_	6.53 +/− 0.67++	0.06 +/− 0.007++	7.500 +/− 0.7++
200 mg/kg LPME + CCl_4_	6.08 +/− 0.30++	0.06 +/− 0.003++	5.33 +/− 0.22 ++
200 mg/kg LPME alone	5.63 +/− 0.20++	0.05 +/− 0.002++	5.50 +/− 1.36++

Mean ± SE (n = 6 number).

** indicate significance from the control group at *P <* 0.01 probability level.

++ indicate significance from the CCl_4_ group at *P <* 0.01 probability level.

### DNA damages (DNA ladder assay; DPA assay)

**Figure 1:**
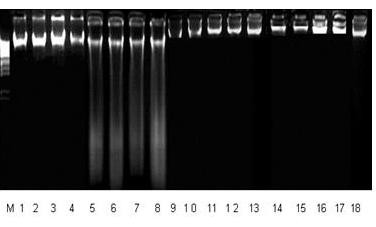
**Agarose gel showing DNA damage by CCl**_**4 **_**and preventive effect of *Launaea procumbens *extracts in different groups.** Lanes (from left) Control (1–4), CCl_4_ (5–8), CCl_4+_ LPME 100 mg/kg b.w., (9–12) CCl_4+_ LPME 200 mg/kg b.w., (13–15), LPME 200 mg/kg b.w., (16–18).

Free radicals of carbon tetrachloride cause testicular DNA fragmentation qualitatively (Figure 1) and quantitatively (Table 5) in testicular tissue. Qualitative analysis revealed that Line (5–8) of DNA printing showed that CCl_4_ causes damages which are absent in control (1–4). Co-administration of 100 mg/kg and 200 mg/kg b.w., LPME in CCl_4_ treated rats cause significant reduction *(p < 0.01)* in DNA damages. Similar observations were found in DPA method.

### Histology of testis in rats and LPME

Histological appearance play important role in study of protective role of LPME in rats. Administration of CCl_4_ caused loss of germ cells, abnormality of germinative epithelium, interruption in meiosis; sperm with abnormal shape and concentration were visible. The ground substance within the interstitium was replaced by fibroblast and inflammatory cells as well as caused atrophy of somniferous tubules. Orally-treatment with LPME revealed a marked repairing of testicular abnormalities induced by CCl_4_ in dose depenent way near to control group (Table 6).

**Table 6:** Effect of LPME on testicular histopathology in rat

**Treatment**	**Somniferous tubules degeneration**	**Meiosis interruption**	**Sperm concentration**	**Germ cell morphology**	**Germinative epithelium**
Control	-	-	-	**-**	-
3 ml/kg CCl_4_	+++	++	+++	++	++
100 mg/kg LPME + CCl_4_	−/+	-	−/+	−/+	−/+
200 mg/kg LPME + CCl_4_	-	-	-	-	−/+
200 mg/kg LPME alone	-	-	-	-	-

-, normal; −/+, mild; ++, medium; +++, severely damaged.

## Discussion

CCl_4_ requires bioactivation by phase I cytochrome P450 system to form reactive metabolic trichloromethyl radical (CCl_3_*) and peroxy trichloromethyl radical (*OOCCl_3_). These free radicals can bind with polyunsaturated fatty acid (PUFA) to produce alkoxy (R*) and peroxy radicals (ROO*), that, in turn, generate lipid peroxides, that are highly reactive, change enzyme activity and finally induce injury or necrosis [5,27]. The injuries induced by CCl_4_ are resulted from free radicals through lipid per oxidation of cell membranes, reduces antioxidant enzyme and antioxidant substrates to induce oxidative stress that is an important factor in acute and chronic injuries in various tissues [28]. *Launaea procumbens* L. possess bioactive ingredients which play important role in reduction of oxidative stress in male albino rats [11]. Testicular oxidative stress appears to be a common feature in infertility, which suggests that, there may be benefits to develop better antioxidant therapies for relevant cases of hypo spermatogenesis [29,30]. The results of the presents study revealed that LPME had significant improvement on body weight, testicular weight and relative tissue weight. The report of Khan and Ahmed [29] revealed significant reduction in weight gain, particularly in studies initiated in male animals, during three months observation of rats receiving carbon tetra chloride in comparison to the controls. The increase in the reproductive organs weights could be due to the increase in lipid peroxidation which was observed in the current study that may be resulted from the oxidative damage induced in rat testes. The amelioration effect of LPME may be due to gallic acid and polyphenolic compounds [16]. Other studies also revealed the importance of herbal extract on testicular tissue [31-33].

CCl_4_ induced marked reduction in sperm count, motility (%), with increase in dead and abnormal sperm count as compared to control group which was significantly restored with both doses of LPME. Previous reports revealed that chemical ingestion cause suppression of sexual behavior of male rats [34] and reductions in motility [35].

Antioxidant enzyme play key role in oxidative infertility. Oxidative stress may result in overproduction of oxygen free-radical precursors and/or decreased efficiency of the antioxidant system. CCl_4_ and oxygen free-radical generation is associated with impaired glutathione metabolism, alterations in the antioxidant status [26]. The results of our present investigation showed that 3 ml/kg CCl_4_ administration in rats caused significant reduction in the activity of antioxidant enzymes, GSH and increased TBARS. Reduction of antioxidant enzymes activity in testicular tissue are might be due to accumulation of free radicals leads to enhanced lipid peroxidation or inactivation of the antioxidative enzymes [36]. Glutathione contents play key role in maintaining antioxidant status. Decrease in GSH activity during CCl_4_ toxicity might be due to the decreased availability of GSH resulted during the enhanced lipid peroxidation. Improvement of testicular GSH levels in rats treated with *Launaea procumbens* extracts in comparison to CCl_4_ administration further demonstrated the antioxidative effect of the plant. Various treatments of *Launaea procumbens* extracts also improved the levels of antioxidant enzymes in CCl_4_ administered rats are due the presence of phenolic and polyphenolic constituents [13] which may have different functional properties such as scavenging of active oxygen species, inhibition of the generation of free radicals and chain breaking activity. Similar observations were also reported with black tea extract on the level of TBARS in rats after CCl_4_ exposure [37]. Jia et al., [8] investigated that oxidative damage can occur in DNA during the peroxidative breakdown of membrane polyunsaturated fatty acids. DNA damage affects homeostasis of various cells leading to induced signal transductions associated with apoptosis and cell proliferation [38]. Administration of *Launaea procumbens* extracts to CCl_4_ intoxicated rats protected and markedly decreased the percentage of fragmented DNA that was also revealed in DNA ladder assay. It may contribute its protective effects by erasing the damaging action of CCl_4_ at DNA level. The protective potential may either involve antioxidant; signal transduction, gene expression, and effective involvement in the metabolic pathways [39].

Histopathalogical study revealed that CCl_4_ treatment showed marked degeneration and alterations of germ cells; however treatment of *Launaea procumbens* showed noticeable improvement in histopathalogical changes induced by CCl_4_ in testis sections. The histological changes in testes of rats administered CCl_4_ are in agreement with Khan and Ahmed [36] who studied the effect of *Digera muricata* against CCl_4_ induced toxicity on the rat's testes. Yousef and Salama [30] reported that oxidative stress results from the production of oxygen radicals in excess of the antioxidant capacity of the stressed tissue. Many conditions or events associated with male infertility are inducers of oxidative stress, which leads to an increase in germ cell apoptosis and subsequent hypospermatogenesis, such stress condition, endocrine signaling, and germ cell apoptosis. Moreover, reactive oxygen species and oxidative damage of bimolecular may contribute to male infertility by reducing sperm function [40]. Minimizing the hazard effects of CCl_4_ by LPME treatment may be due to the flavoniods in LPME, which exert many health-promoting effects, including the ability to increase intercellular antioxidant levels, decrease capillary permeability and fragility and scavenge oxidants and free radicals [41,42].

## Conclusion

This study provided the scientific proof that LPME is useful remedy for oxidative stress and reproductive hormonal dysfunction in male. Further work towards the isolation of bioactive constituents responsible for these activities is in progress in our lab.

## Competing interests

The authors declare that they have no competing interests.

## Authors’ contributions

RAK made significant contribution to acquisition of data, analysis, drafting of the manuscript. The author read and approved the final manuscript.
